# Single-cell and spatial transcriptomic profiling reveals the expression characteristics of PTPRR in epithelial cells and its potential implications in pancreatic cancer metastasis

**DOI:** 10.3389/fimmu.2026.1843505

**Published:** 2026-05-13

**Authors:** Runyang Li, Xuechun Xu, Wenqing Zhang, Yan-yan Zhan

**Affiliations:** Cancer Research Center, School of Medicine, Xiamen University, Xiamen, Fujian, China

**Keywords:** immunosuppression, pancreatic cancer, PTPRR, ScRNA-seq, tumor microenvironment

## Abstract

**Background:**

Pancreatic ductal adenocarcinoma (PDAC) is a highly malignant digestive tumor with an extremely low 5-year survival rate, and KRAS mutation is its predominant molecular characteristic. Protein tyrosine phosphatase receptor R (PTPRR) functions as a tumor suppressor in most cancers; however, its expression pattern, biological function, and regulatory mechanism in PDAC remain largely unknown.

**Methods:**

Multiple public datasets including TCGA-PAAD and GSE79668 were integrated for differential gene screening. Survival analysis, TMB evaluation, KEGG enrichment, single-cell and spatial transcriptomic analyses were performed. *In vitro* functional assays (MTT, Transwell) and plasmid transfection were applied to validate the role of PTPRR in PDAC malignant phenotypes.

**Results:**

PTPRR was significantly upregulated in PDAC tissues and almost exclusively expressed in pancreatic ductal epithelial cells, with significantly higher levels in metastatic lesions than in primary tumors. High PTPRR expression correlated with lymph node metastasis, advanced stage, and poor prognosis (Log-rank P<0.05), serving as an independent adverse prognostic factor. PTPRR-related genes were enriched in MAPK and RAS signaling. The PTPRR high-expression group exhibited higher TMB (P = 0.0035) and increased mutation frequencies of KRAS and TP53. PTPRR was highly expressed in metastatic PDAC cell lines; its overexpression promoted migration and invasion, while knockdown suppressed proliferation, migration, and invasion. At single-cell level, PTPRR-high epithelial cells showed elevated stemness and formed a specific niche with iCAFs via EDN, EGF, and PDGF signaling. In metastatic patients, PTPRR-high tumor cells were associated with an immunosuppressive microenvironment by interacting with T cells and other immune cells through TGFβ, CCL, and CXCL pathways.

**Conclusion:**

PTPRR serves as an epithelial-specific biomarker closely associated with PDAC progression. It is correlated with PDAC metastasis, potentially via regulating the MAPK pathway, interacting with iCAFs, and associating with an immunosuppressive microenvironment in a metastasis-dependent manner. KRAS mutation may underlie its functional conversion from tumor suppressor to oncogene, which remains a hypothetical mechanism. PTPRR represents a promising prognostic biomarker and potential therapeutic target for PDAC.

## 1 Introduction

Pancreatic cancer is one of the deadliest malignancies worldwide, with pancreatic ductal adenocarcinoma (PDAC) accounting for more than 90% of all pancreatic cancer cases. It has an insidious onset and rapid progression, and most patients are diagnosed at an advanced stage, with a surgical resection rate of only 10%–20% and a 5−year survival rate of less than 13% (1, 2). The highly malignant phenotype of pancreatic cancer is closely linked to complex molecular regulatory disorders. Approximately 90% of pancreatic cancer patients harbor KRAS gene mutations, and the sustained activation of mutant KRAS drives abnormal activation of oncogenic signaling pathways such as the mitogen−activated protein kinase (MAPK) pathway, promoting tumor cell proliferation, invasion, and metastasis as a core driver of pancreatic cancer carcinogenesis and progression (3). Currently, the clinical treatment of pancreatic cancer mainly relies on chemotherapy and surgery, lacking precise and effective targeted therapeutic strategies. Therefore, in−depth exploration of key regulatory genes in pancreatic cancer development and clarification of their molecular mechanisms are of great clinical significance for developing novel diagnostic biomarkers and therapeutic targets (4).

Protein tyrosine phosphatases (PTPs) are important signaling regulatory molecules that participate in various physiological and pathological processes including cell proliferation, differentiation, apoptosis, and signal transduction via dephosphorylating protein tyrosine residues, and their dysfunction is closely associated with tumorigenesis and progression (5, 6). Protein tyrosine phosphatase receptor type R (PTPRR), a key member of the PTP family, has been confirmed to function as a tumor suppressor in multiple malignant tumors such as cervical cancer, ovarian cancer, and lung cancer. Its silenced expression leads to excessive activation of the MAPK signaling pathway, thereby fueling tumor progression (6–9). Existing studies have shown that PTPRR can inhibit abnormal activation of the MAPK pathway by dephosphorylating ERK1/2 proteins, thus suppressing the malignant proliferation of tumor cells (9). However, current research on the expression pattern, regulatory mechanism, and clinical prognostic correlation of the PTPRR gene in pancreatic cancer remains incomplete, and whether it participates in the malignant progression of pancreatic cancer by regulating the KRAS−MAPK signaling pathway requires further exploration (10).

With the development of bioinformatics technologies, multi−omics integrated analysis (including bulk transcriptomics, single−cell transcriptomics, and spatial transcriptomics) has become a powerful tool for dissecting tumor molecular mechanisms, enabling systematic characterization of gene expression patterns, cell subset distribution, and spatial interactions at the global, cellular, and spatial levels (11–13). In this study, we systematically investigated the expression pattern, clinical significance, and potential mechanism of PTPRR in pancreatic cancer by integrating multiple public datasets including TCGA and GEO, combined with single−cell and spatial transcriptomic analyses (14, 15). This study aims to clarify the oncogenic role of PTPRR in pancreatic cancer and its underlying molecular mechanisms, providing a novel theoretical basis and potential targets for prognostic evaluation and molecular targeted therapy of pancreatic cancer.

## 2 Methods

### 2.1 Dataset source and preprocessing

Public datasets used in this study were obtained from the Gene Expression Omnibus (GEO) and The Cancer Genome Atlas (TCGA), specifically including: the TCGA−PAAD dataset (clinical information and transcriptomic data of pancreatic cancer), the GSE79668 dataset (transcriptomic data related to pancreatic cancer tumor staging), the GSE263733 dataset (single−cell transcriptomic data of primary and metastatic pancreatic cancer samples), a validation dataset for pancreatic cancer single−cell transcriptomics, the GSE235449 dataset (pancreatic cancer spatial transcriptomic data), and the GSE194247 dataset (pancreatic cancer single−cell transcriptomic data) (14–17). In addition, pancreatic cancer cell line expression data were derived from the Cancer Cell Line Encyclopedia (CCLE).

All transcriptomic data were subjected to standardized preprocessing. Differential expression analysis was performed using the DESeq2 package in R. Single−cell transcriptomic data were quality−controlled using the Seurat software (v4.0.5), with cells filtered for mitochondrial gene ratio >10%, gene count <200, or gene count >6000, followed by normalization and scaling. Cell clustering was achieved via PCA dimensionality reduction and UMAP embedding. Spatial transcriptomic data were processed with the Seurat software to remove batch effects and map cell subsets, ensuring data reliability.

### 2.2 Bioinformatic analysis

#### 2.2.1 Differential expression analysis

Differentially expressed genes (DEGs) were screened uniformly using the DESeq2 package in R. Specifically, differential expression analyses of “lymph node metastasis group vs. non−lymph node metastasis group” and “tumor stage−related (corresponding to GSE79668 staging)” were first performed in the TCGA−PAAD dataset, followed by intersection analysis of the two gene sets from these differential analyses. Meanwhile, differential expression analyses of “lymph node metastasis group vs. non−lymph node metastasis group” and “T2 stage vs. T3 stage” were conducted in the GSE79668 dataset, and the intersection of the two gene sets was obtained. All differential analyses used |log_2_(fold change, FC)| > 1 and adjusted P−value (Padj) < 0.05 as screening criteria. Finally, a second intersection analysis was performed between the gene sets from the TCGA−PAAD and GSE79668 datasets to identify potential candidate genes.

#### 2.2.2 Survival analysis

Based on clinical follow−up information from the TCGA−PAAD dataset and using the GEPIA database, patients were divided into high− and low−expression groups according to the median PTPRR expression level, and grouped by clinical characteristics (lymph node metastasis, tumor volume). Survival curves were plotted using the Kaplan−Meier method, and the log−rank test was used to analyze differences in overall survival between groups, with P < 0.05 considered statistically significant.

#### 2.2.3 Pathway enrichment and tumor mutational burden analysis

Kyoto Encyclopedia of Genes and Genomes (KEGG) pathway enrichment analysis of DEGs was performed using the clusterProfiler package in R, with P < 0.05 indicating significant enrichment. TMB values (number of mutations per megabase) for each sample were calculated using somatic mutation data from TCGA−PAAD. The Wilcoxon rank−sum test was used to compare TMB differences between the PTPRR high− and low−expression groups. Mutation frequencies of hotspot genes such as KRAS and TP53 in the two groups were counted, and the chi−square test was used to analyze intergroup differences.

### 2.3 Cellular experiments

#### 2.3.1 Cell lines and culture conditions

Pancreatic cancer cell lines used in this study included primary pancreatic cancer cell lines (MIA PaCa−2, PANC−1) and metastatic pancreatic cancer cell lines (CFPAC−1, Capan−1), all purchased from the Cell Bank of the Chinese Academy of Sciences. All cells were cultured in DMEM medium supplemented with 10% fetal bovine serum and 1% penicillin−streptomycin double antibody, and routinely maintained in a constant−temperature incubator at 37 °C with 5% CO_2_. Cells were passaged regularly to ensure logarithmic growth for subsequent experiments.

#### 2.3.2 Cell transfection and efficiency verification

PTPRR overexpression plasmids (PLVX−PTPRR) and knockdown plasmids (PLKO−shPTPRR) were constructed. Two specific shRNA primers with distinct target sites were used for the knockdown plasmids to ensure knockdown efficiency, with empty vector (PLVX) and negative control knockdown vector (PLKO−shNC) as controls. The sequences of PTPRR knockdown primers were as follows:

Hs−shPTPRR−1−F: CCGGTCATTGAGCACCTACATTAATCTCGAGATTAATGTAGGTGCTCAATGATTTTTG;

Hs−shPTPRR−1−R: AATTCAAAAATCATTGAGCACCTACATTAATCTCGAGATTAATGTAGGTGCTCAATGA;

Hs−shPTPRR−2−F: CCGGCACCTATCGCCCATCACATTACTCGAGTAATGTGATGGGCGATAGGTGTTTTTG;

Hs−shPTPRR−2−R: AATTCAAAAACACCTATCGCCCATCACATTACTCGAGTAATGTGATGGGCGATAGGTG.

Plasmids were transfected into PANC−1 and CFPAC−1 cells using Lipofectamine 3000 transfection reagent according to the manufacturer’s instructions. At 48 h post−transfection, quantitative real−time polymerase chain reaction (qRT−PCR) was performed to detect PTPRR mRNA expression and verify transfection efficiency. All experiments were repeated independently three times.

#### 2.3.3 *In vitro* functional assays

Cell proliferation was measured using the MTT assay. Transfected cells were seeded into 96−well plates, and MTT reagent (5 mg/mL) was added at 0, 24, 48, and 72 h. After 4 h of incubation, the supernatant was discarded, 150 μL dimethyl sulfoxide (DMSO) was added to each well, and the absorbance at 570 nm was measured using a microplate reader after shaking to dissolve formazan crystals, followed by calculation of the cell proliferation rate. Cell migration and invasion were assessed using Transwell assays. Matrigel was omitted for migration assays but pre−coated for invasion assays. Transfected cells were seeded into the upper Transwell chambers, and medium containing 10% fetal bovine serum was added to the lower chambers. After 24 h of culture, cells were fixed and stained, and the number of transmembrane cells was counted under a microscope for quantitative analysis of migration and invasion. All experiments were repeated independently three times. Data are presented as the mean ± standard deviation ( $\bar{x}$ ± s), and intergroup differences were analyzed using the t−test, with P < 0.05 considered statistically significant.

### 2.4 Single-cell transcriptomic analysis

Single-cell transcriptomic data from GSE26373, GSE154778, GSE235449, and GSE194247 were analyzed using the Seurat software. After cell quality control and normalization, highly variable genes were identified using the FindVariableFeatures function. Following PCA dimensionality reduction, cell clustering was performed using the FindNeighbors and FindClusters functions, and cell clusters were visualized via UMAP. Cell annotation was performed using the SingleR package with the HumanPrimaryCellAtlasData reference dataset (saved as ref_Human_all.RData). Cell subsets were further verified and defined using canonical cell-type specific marker genes as follows: B cells (*CD79A*, *MS4A1*), Dendritic cells (DC; *CD1C*, *FCER1A*, *CD1E*), T cells (*CD3E*, *CD4*, *CD8A*, *CD8B*), Endothelial cells (*VWF*), Epithelial cells (*KRT19*, *PTPRR*), Fibroblasts (*COL1A1*, *COL1A2*), Monocytes (*S100A8*, *FCN1*), Schwann cells (*SOX10*), and Stellate cells (*RGS5*). Cell stemness was analyzed using the CytoTRACE2 tool to calculate stemness scores and evaluate stemness levels. Pseudotime analysis was performed using Monocle3 software to construct developmental trajectories of ductal or epithelial cells, with the most stem−like cells selected as the developmental starting point to fit cell differentiation trajectories. The FindMarkers function in Seurat was used to screen DEGs between PTPRR−high cells and other cells, and KEGG pathway enrichment analysis was performed to explore molecular mechanisms. The proportion of PTPRR−positive cells in each sample was counted, and samples with a positive rate >10% were defined as PTPRR−high samples.

### 2.5 Spatial transcriptomic analysis

For the GSE235449 spatial transcriptomic dataset, annotated single−cell data were first mapped to spatial transcriptomic slices using the Seurat software to achieve spatial localization of cell subsets. CAFs were subdivided into iCAF, myCAF, and apCAF subsets using a gene set scoring method based on CAF marker gene sets.

#### 2.5.1 Cellular niche analysis

Based on the cellular composition ratio of each spatial transcriptomic spot, cellular niches were identified using the Seurat package in R and NicheNet software, and the spatial co−localization of PTPRR−high epithelial cells with other cell types (especially iCAFs) was analyzed.

#### 2.5.2 Molecular niche analysis

After batch effect removal, molecular niches were identified based on the gene expression profile of each spot. KEGG pathway enrichment analysis was performed on differential molecular niches to explore cell−cell communication−related pathways.

### 2.6 Cell-cell communication analysis

Cell−cell communication analysis of single−cell transcriptomic data was performed using CellChat software (v1.1.3). For the GSE194247 dataset, focus was placed on the PTPRR−high epithelial cell subset to analyze the strength of signal communication, core pathways, and ligand−receptor pairs with other cell clusters (especially CAFs). For the GSE263733 dataset, samples were divided into high− and low−expression groups based on PTPRR expression levels. Overall cell−cell communication differences between the two groups were compared, with emphasis on immunosuppressive pathways (TGFβ, CCL, CXCL, etc.) and ligand−receptor pairs to characterize differences in the tumor immune microenvironment. Heatmaps and circle plots were used to visualize cell−cell communication results and screen core signaling pathways and ligand−receptor pairs.

### 2.7 Statistical analysis

All experimental data and bioinformatic analyses were statistically processed using R software (v4.1.2). Measurement data are presented as the mean ± standard deviation ( $\bar{x}$ ± s), and intergroup differences were analyzed using the t−test or Wilcoxon rank−sum test. Enumeration data were analyzed using the chi−square test. Survival analysis was performed using the Kaplan−Meier method and log−rank test. Pathway enrichment analysis was conducted using Fisher’s exact test. P < 0.05 was considered statistically significant.

## 3 Results

### 3.1 PTPRR is highly expressed in pancreatic cancer and correlates with poor prognosis and high tumor mutational burden

**Figure 1:**
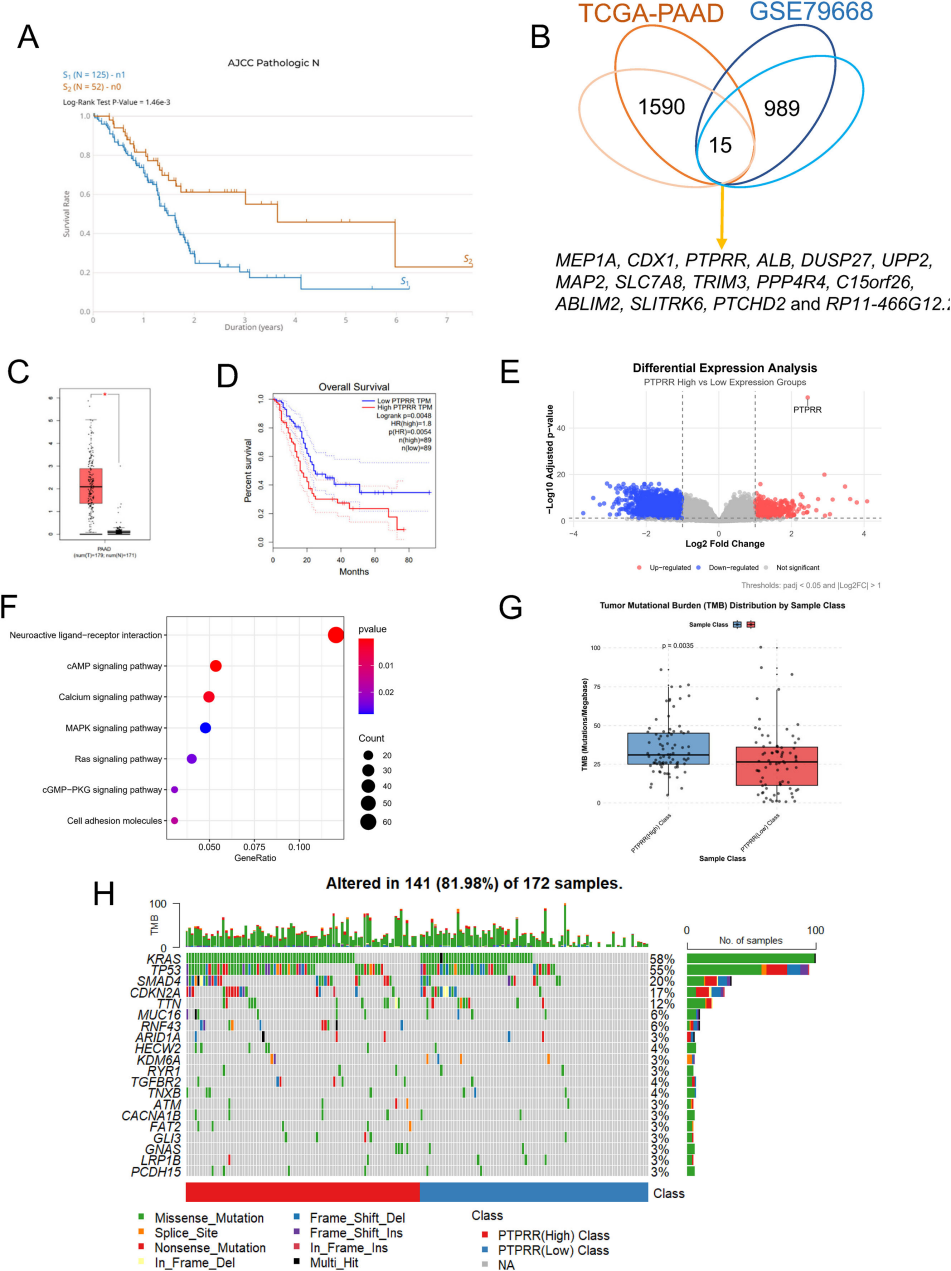
Expression, prognostic value, related pathways and TMB analysis of PTPRR in pancreatic cancer. **(A)** Kaplan−Meier survival curves for overall survival of pancreatic cancer patients with or without lymph node metastasis based on the TCGA−PAAD dataset (Log−rank P = 0.00146). **(B)** Venn diagram of overlapping differentially expressed genes between “lymph node metastasis vs. non−lymph node metastasis” in the TCGA−PAAD dataset and “T2 stage vs. T3 stage” in the GSE79668 dataset, with a total of 15 potential candidate genes identified. **(C)** Comparison of PTPRR expression levels between pancreatic cancer tissues and normal pancreatic tissues in the TCGA−PAAD dataset (P<0.01). **(D)** Kaplan−Meier survival curves for overall survival of pancreatic cancer patients with high vs. low PTPRR expression in the TCGA−PAAD dataset (Log−rank P<0.05). **(E)** Volcano plot of differentially expressed genes between PTPRR high- and low-expression groups in TCGA pancreatic cancer (PAAD) patients. **(F)** KEGG pathway enrichment analysis of differentially expressed genes between PTPRR high− and low−expression groups, showing significant enrichment in cAMP, MAPK, RAS and other signaling pathways. **(G)** Comparison of tumor mutational burden (TMB) levels between PTPRR high− and low−expression groups in the TCGA−PAAD dataset (P = 0.0035). **(H)** Comparison of KRAS and TP53 mutation frequencies between PTPRR high− and low−expression groups in the TCGA−PAAD dataset.

Survival analysis based on the TCGA−PAAD dataset showed that overall survival was significantly shorter in pancreatic cancer patients with lymph node metastasis than in those without (log−rank P = 0.00146, **Figure 1A**), and patients with larger tumor volume also had poorer overall survival (**Supplementary Figure 1A**), indicating that lymph node metastasis and tumor volume are important prognostic factors in pancreatic cancer. To screen key genes associated with malignant progression of pancreatic cancer, differential expression analyses of “lymph node metastasis group vs. non−lymph node metastasis group” in TCGA−PAAD and “T2 stage vs. T3 stage” in GSE79668 were performed, and the intersection of the two DEG sets yielded 15 potential candidate genes (**Figure 1B**).

Further single−gene expression analysis revealed that PTPRR expression was significantly higher in pancreatic cancer tissues than in normal pancreatic tissues (P < 0.01, **Figure 1C**). Among the 15 candidate genes, PTPRR was the only one that was both significantly highly expressed in pancreatic cancer and associated with poor prognosis (Log-rank P<0.05, **Figure 1D**; **Supplementary Figure 4**), suggesting a core regulatory role in malignant progression of pancreatic cancer.

The TCGA−PAAD cohort was divided into PTPRR high− and low−expression groups, and 1243 DEGs were identified (|log_2_FC| > 1, Padj < 0.05) (P < 0.01, **Figure 1E**). KEGG pathway enrichment analysis showed that these DEGs were significantly enriched in cAMP, MAPK, and RAS signaling pathways (**Figure 1F**). Enrichment of the MAPK pathway suggested that PTPRR may participate in pancreatic cancer malignant progression by regulating this pathway.

TMB analysis showed that TMB was significantly higher in the PTPRR high−expression group than in the low−expression group (P = 0.0035, **Figure 1G**). Analysis of frequently mutated genes revealed that the mutation frequencies of KRAS and TP53 were significantly higher in the PTPRR high−expression group (**Figure 1H**), providing important clues for subsequent exploration of the molecular mechanism underlying PTPRR functional conversion.

### 3.2 PTPRR is highly expressed in metastatic pancreatic cancer cells and regulates malignant phenotypes

**Figure 2:**
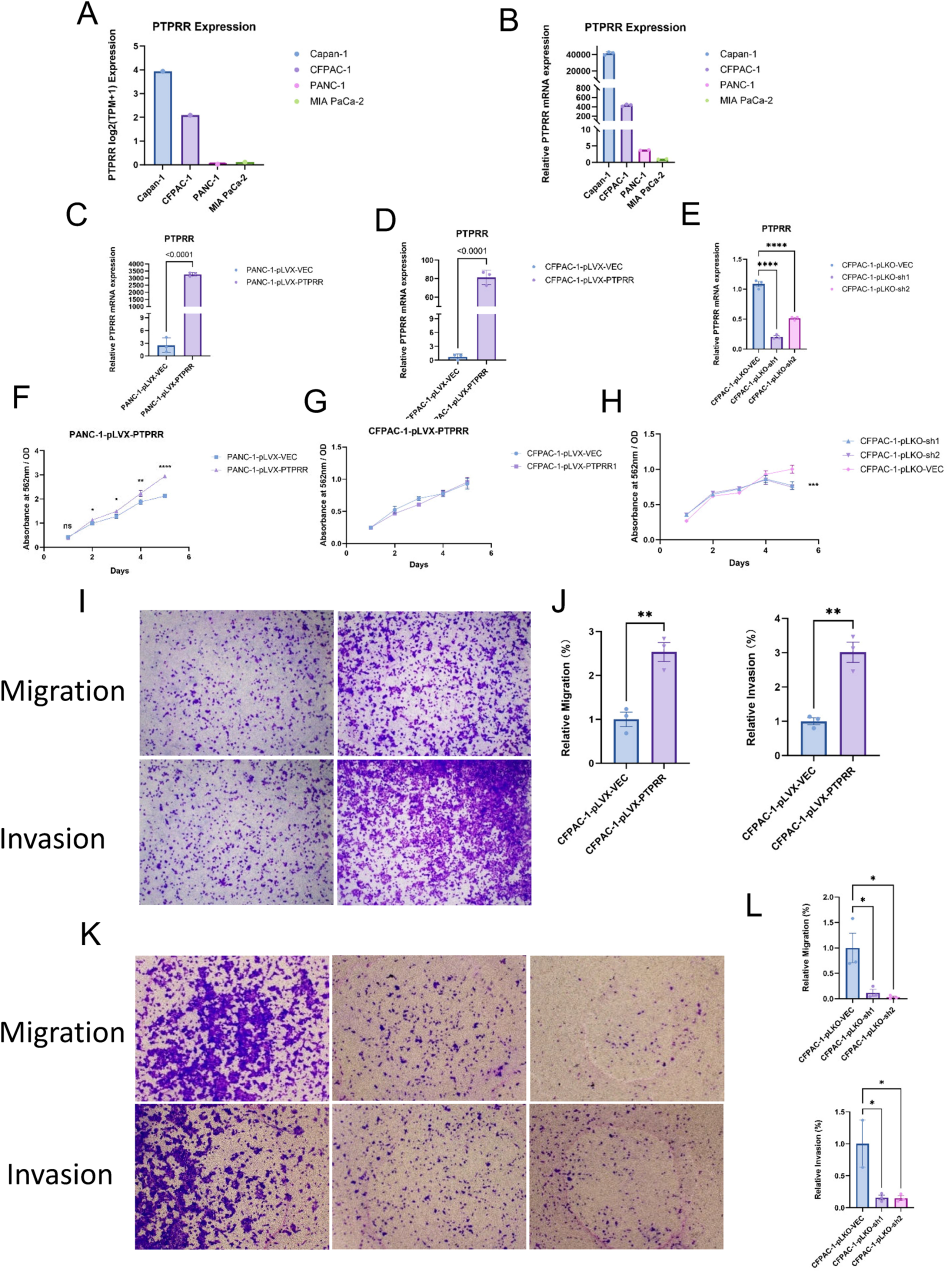
Expression of PTPRR in pancreatic cancer cells and its regulatory effects on malignant phenotypes. **(A)** Comparison of PTPRR expression levels between metastatic pancreatic cancer cell lines (CFPAC−1, Capan−1) and primary pancreatic cancer cell lines (MIA PaCa−2, PANC−1) in the CCLE database. **(B)** Validation of PTPRR mRNA and protein expression levels in CFPAC−1, Capan−1 versus MIA PaCa−2, PANC−1 cells in our laboratory (consistent with database results). **(C)** Validation of PTPRR overexpression efficiency and detection of cell proliferative ability after PTPRR overexpression in PANC−1 cells (P<0.001). **(D)** Validation of PTPRR overexpression efficiency in CFPAC−1 cells. **(E)** Validation of PTPRR knockdown efficiency in CFPAC−1 cells. **(F)** Quantitative analysis of proliferative ability in PANC−1 cells after PTPRR overexpression (P<0.001). **(G)** Quantitative analysis of proliferative ability in CFPAC−1 cells after PTPRR overexpression (P = 0.402, no significant difference). **(H)** Quantitative analysis of proliferative ability in CFPAC−1 cells after PTPRR knockdown (P<0.001). **(I, J)** Detection and quantitative analysis of migratory and invasive abilities in CFPAC−1 cells after PTPRR overexpression (P<0.001). **(K, L)** Detection and quantitative analysis of migratory and invasive abilities in CFPAC−1 cells after PTPRR knockdown (P<0.001).

Based on the association of PTPRR with pancreatic cancer metastasis and prognosis indicated by bulk transcriptomic data, we further verified its regulatory role in pancreatic cancer malignant phenotypes via cellular experiments. CCLE database analysis showed that PTPRR expression was significantly higher in metastatic pancreatic cancer cell lines (CFPAC−1, Capan−1) than in primary cell lines (MIA PaCa−2, PANC−1, **Figure 2A**). *In vitro* experimental results in our laboratory were consistent with database analysis: PTPRR mRNA and protein expression were markedly higher in CFPAC−1 and Capan−1 cells than in MIA PaCa−2 and PANC−1 cells (**Figure 2B**), indicating a close correlation between PTPRR expression and pancreatic cancer metastatic potential.

To clarify the regulatory effect of PTPRR on pancreatic cancer malignant phenotypes, *in vitro* functional assays were performed. Overexpression of PTPRR in PANC−1 primary cells (**Figure 2C**) significantly enhanced cell proliferation (P < 0.001, **Figures 2C, F**). PTPRR overexpression in CFPAC−1 metastatic cells had no significant effect on proliferation (P = 0.402, **Figures 2D, G**) but markedly promoted cell migration and invasion (P < 0.001, **Figures 2I, J**). In contrast, PTPRR knockdown in CFPAC−1 cells (**Figure 2E**) significantly inhibited cell proliferation (**Figure 2H**, P < 0.001), migration, and invasion (**Figures 2K, L**, P < 0.001). These results confirm that PTPRR critically regulates the malignant phenotypes of pancreatic cancer cells, particularly promoting metastatic potential.

### 3.3 Single-cell expression characteristics and stemness analysis of PTPRR in pancreatic cancer

**Figure 3:**
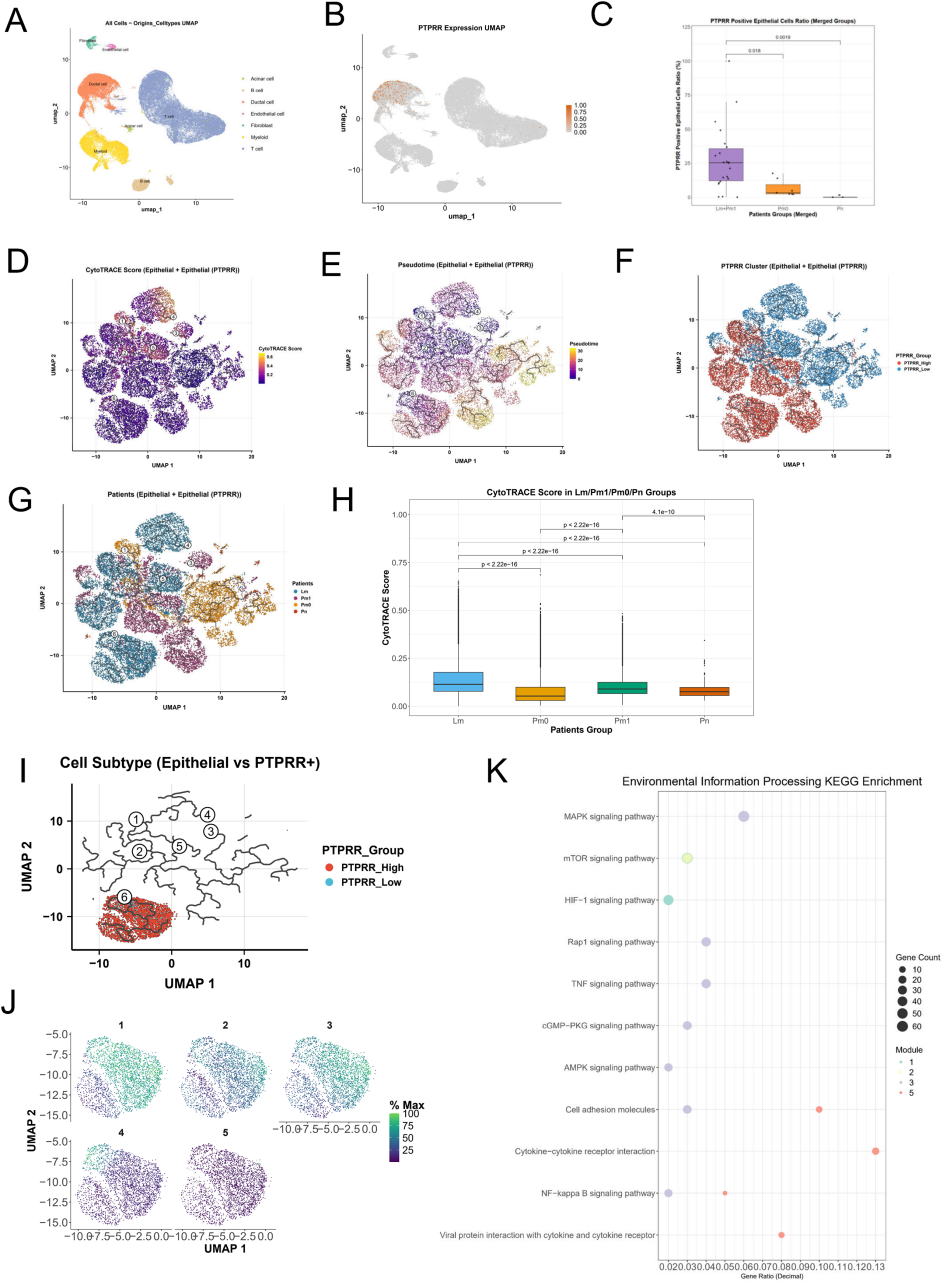
Single−cell expression characteristics and stemness analysis of PTPRR in pancreatic cancer. **(A)** UMAP visualization of cell clustering in the GSE263733 single−cell dataset containing primary and metastatic pancreatic cancer samples. **(B)** Expression distribution of PTPRR in each cell type in the GSE263733 dataset, showing predominant high expression in ductal epithelial cells (tumor cells). **(C)** Comparison of PTPRR expression levels among primary pancreatic cancer samples (Pm0), liver metastasis samples (Lm) and other distant metastasis samples (Pm1) in the GSE263733 dataset. **(D)** Visualization of CytoTRACE2 stemness scores of ductal cells in the GSE263733 dataset. **(E)** Developmental trajectory of ductal cells constructed by Monocle3, with the 6 most stem−like cells selected as the developmental starting points. **(F)** Distribution characteristics of ductal cells from PTPRR high− and low−expression groups along the developmental trajectory. **(G)** Distribution characteristics of ductal cells from liver metastasis, other distant metastasis, primary tumor and paracancerous tissues along the developmental trajectory. **(H)** Statistical analysis of stemness scores of ductal cells in the four groups, showing higher stemness in metastatic samples than in primary samples, with the highest stemness in liver metastasis samples. **(I)** Differential gene expression trajectory analysis of the developmental trajectory region harboring PTPRR−high ductal cells. **(J)** Five gene expression developmental modules identified based on the developmental trajectory. **(K)** KEGG pathway enrichment analysis of five gene modules constructed based on the developmental trajectory of epithelial cells.

To further define the expression localization, cell specificity, and association with metastasis of PTPRR, we analyzed the GSE263733 single−cell dataset containing primary and metastatic pancreatic cancer samples (**Figure 3A**; **Supplementary Figure 1B**). PTPRR was almost exclusively highly expressed in pancreatic ductal epithelial cells (tumor cells), with extremely low expression in immune cells, fibroblasts, and other cell types (**Figure 3B**), confirming that pancreatic ductal epithelial cells are the primary cellular source of PTPRR. This finding was validated in the pancreatic cancer dataset GSE154778 (**Supplementary Figures 1C, D**).

Grouped analysis showed that PTPRR expression was significantly lower in primary pancreatic cancer samples (Pm0) than in liver metastasis (Lm) and other distant metastasis (Pm1) samples (**Figure 3C**), consistent with previous bulk transcriptomic and cellular data and further supporting the correlation between high PTPRR expression and pancreatic cancer metastasis.

We quantified the percentage of PTPRR-positive cells in all samples and defined the PTPRR-high group as those with a positive cell percentage >10% — this cutoff was chosen given that the maximum percentage of PTPRR-positive cells in primary tumors was approximately 7%, ensuring no false-positive classification of primary tumors. To verify the robustness of this cutoff, we performed threshold sensitivity analysis using gradient cutoffs of 5%, 10%, 15%, and 20%. Among these, the 5% and 10% cutoffs yielded statistically significant differences in grouping (P<0.05), whereas the 15% and 20% cutoffs failed to show significant differences due to the reduced number of PTPRR-high samples. Considering the balanced statistical power, strong biological relevance, and ability to avoid primary tumor contamination, the 10% cutoff was designated as the optimal threshold for subsequent analyses (**Supplementary Figure 1E**). To explore the differentiation characteristics and malignant potential of cells in the PTPRR high−expression group, all ductal cells were extracted for stemness scoring and pseudotime analysis. Stemness scores were calculated using CytoTRACE2 (**Figure 3D**), and Monocle3 was used to construct developmental trajectories of ductal cells with the six most stem−like cells as the starting point (**Figure 3E**). Ductal cells from the PTPRR high− and low−expression groups exhibited distinct distribution patterns along the trajectory (**Figure 3F**), and ductal cells from liver metastasis, other distant metastasis, primary tumor, and paracancerous tissues also showed significant separation (**Figure 3G**).

Stemness score analysis revealed that metastatic samples had higher overall stemness than primary samples, with liver metastasis samples showing the highest stemness (**Figure 3H**). Further analysis showed that trajectory region 6, one of the developmental starting points, was almost exclusively occupied by PTPRR−high ductal cells. Gene expression trajectory differential analysis of this region identified five gene expression developmental modules (**Figures 3I, J**). KEGG enrichment analysis showed that DEGs in Module 3 were significantly enriched in the MAPK signaling pathway, suggesting that PTPRR may confer enhanced malignant potential to ductal epithelial cells by activating the MAPK pathway (**Figure 3K**).

### 3.4 High PTPRR expression correlates with spatial distribution and tumor microenvironment characteristics in pancreatic cancer

**Figure 4:**
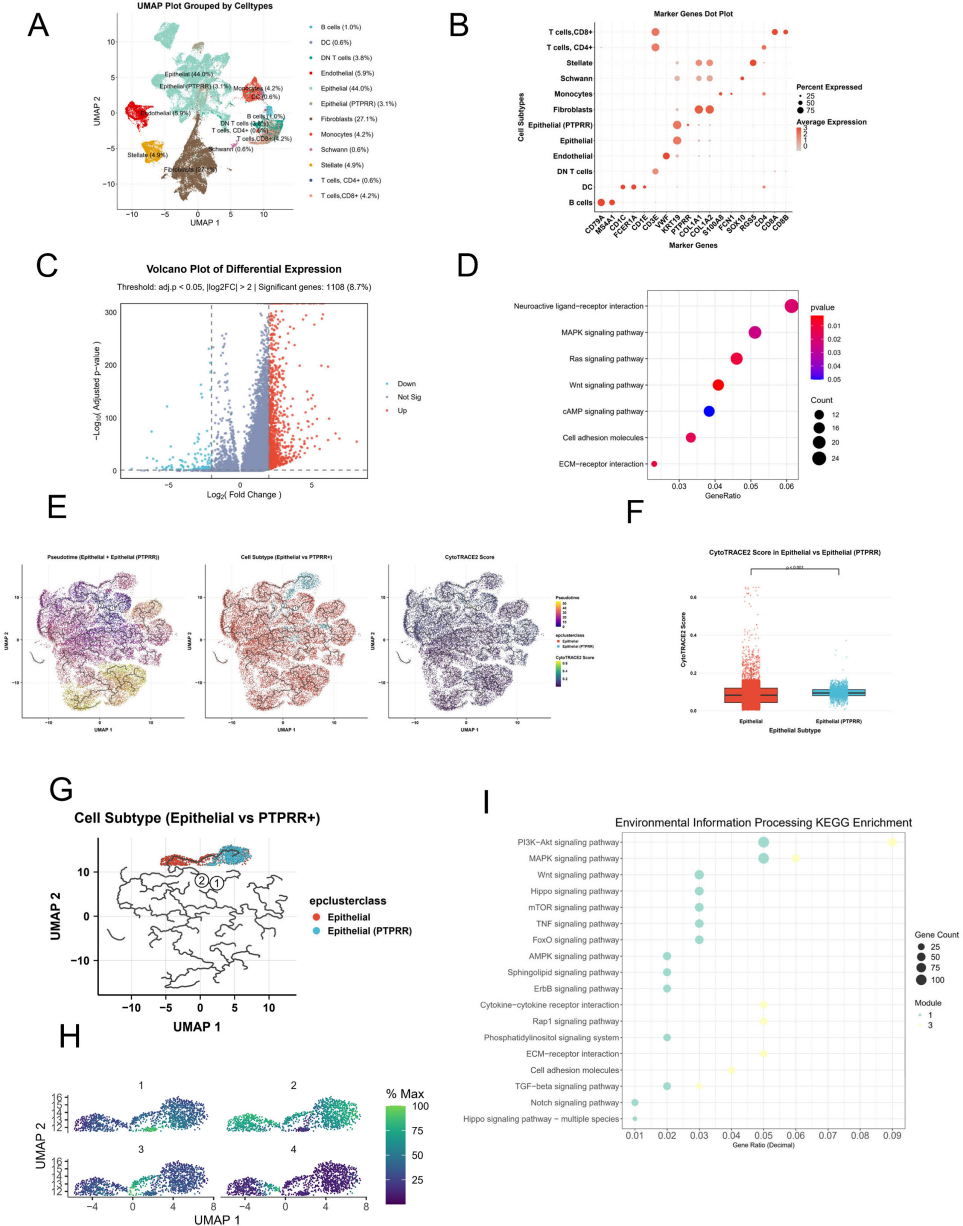
High PTPRR expression in association with stemness of pancreatic cancer epithelial cells and related pathway analysis. **(A)** Cell clustering and annotation results of the GSE235449 single−cell dataset, with a total of 12 cell types identified. **(B)** Bubble plot of marker genes for cell annotation. **(C)** Volcano plot of differentially expressed genes between PTPRR−high epithelial cells and other epithelial cells, with 1108 differentially expressed genes screened. **(D)** KEGG pathway enrichment analysis of differentially expressed genes between PTPRR−high epithelial cells and other epithelial cells, showing enrichment in MAPK, RAS, cAMP and other pathways. **(E)** Stemness scores of epithelial cells and developmental trajectory constructed by Monocle3 in the GSE235449 dataset. **(F)** Comparison of stemness scores between PTPRR−high epithelial cells and other epithelial cells. **(G)** Distribution characteristics of the PTPRR−high epithelial cell subset along the developmental trajectory. **(H)** Four gene modules associated with developmental programs constructed based on developmental trajectory analysis. **(I)** KEGG pathway enrichment analysis of four gene modules constructed based on the developmental trajectory of epithelial cells, with Modules 1 and 3 mainly enriched in MAPK, PI3K−AKT and other pathways.

To spatially characterize the distribution of PTPRR−high epithelial cells and their interactions with the tumor microenvironment, we analyzed the GSE235449 dataset with matched single−cell and spatial transcriptomic data. Twelve distinct cell types were identified via single-cell clustering and annotation (**Figures 4A, B**). The full list of marker genes used for cell annotation is provided in **Supplementary Table 1**. Given the generally low PTPRR expression in primary pancreatic cancer, a PTPRR−high epithelial cell subset was identified via clustering (**Supplementary Figures 1F, G**).

Differential analysis between PTPRR−high epithelial cells and other epithelial cells identified 1108 DEGs (**Figure 4C**). KEGG enrichment analysis showed that these DEGs were also enriched in MAPK, RAS, and cAMP signaling pathways (**Figure 4D**), consistent with bulk transcriptomic results and confirming the MAPK pathway as a key mediator of PTPRR−driven pancreatic cancer progression.

Stemness and developmental trajectory analysis of all epithelial cells showed that PTPRR−high epithelial cells had slightly higher stemness than other epithelial cells (**Figure 4F**), although the most stem−like cells were conventional epithelial cells (**Figure 4E**). Developmental trajectories were constructed with the most stem−like cells as the starting point, focusing on the PTPRR−high epithelial subset (**Figures 4G, H**). Four gene modules were built along the trajectory, and DEGs in Modules 1 and 3 were significantly enriched in MAPK and PI3K−AKT pathways (**Figure 4I**). This suggests gradual activation of the MAPK pathway during the differentiation of conventional epithelial cells into PTPRR−high epithelial cells, with sustained activation following metastasis.

**Figure 5:**
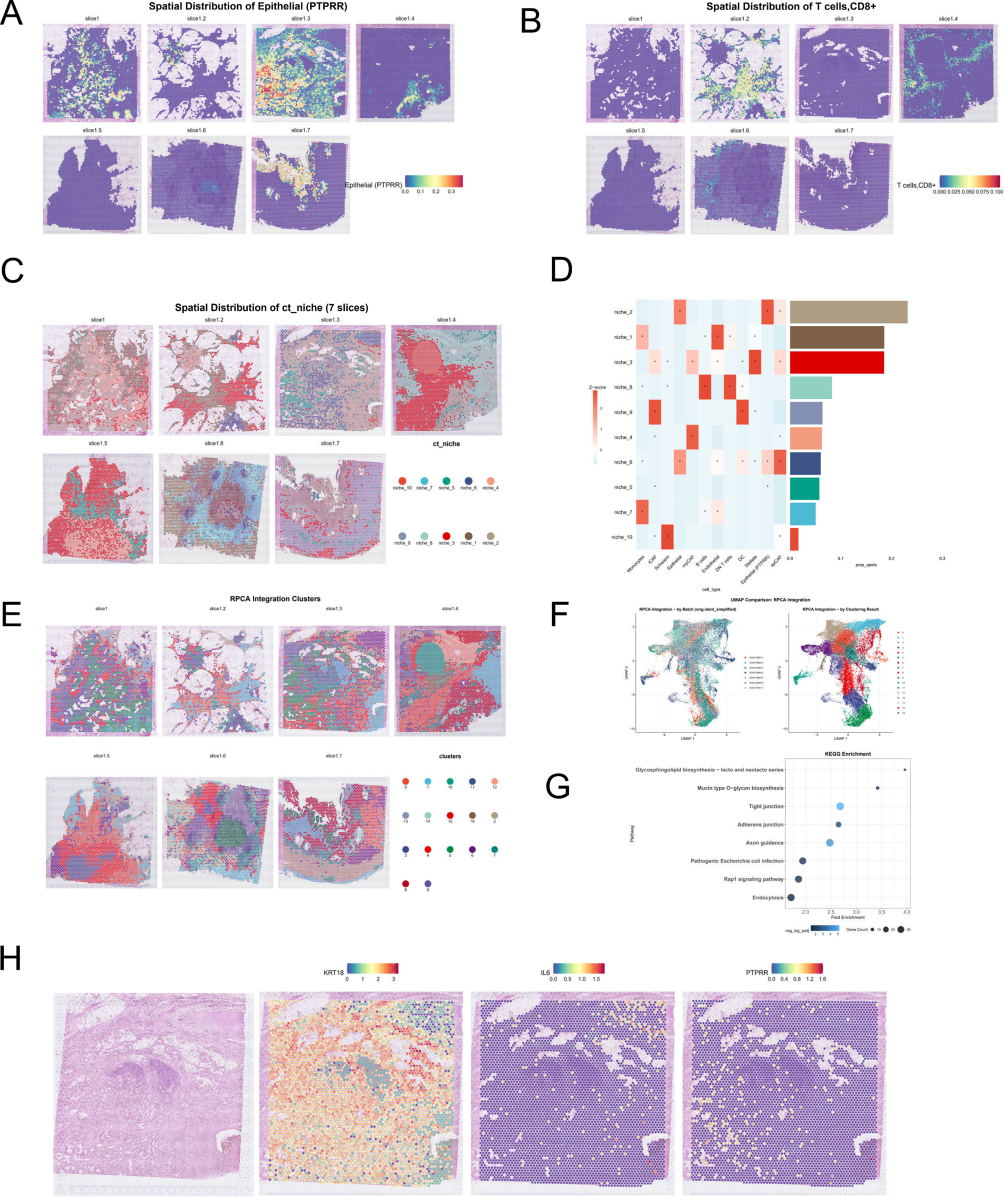
Spatial distribution of PTPRR−high epithelial cells and their association with the tumor microenvironment. **(A)** Distribution and proportion statistics of PTPRR−high epithelial cells in GSE235449 spatial transcriptomic slices, with significantly higher proportions in slices 1, 3 and 7 than in other slices (maximum proportion ≤0.4). **(B)** Infiltration distribution of CD8^+^ T cells in GSE235449 spatial transcriptomic slices, showing almost no CD8^+^ T−cell infiltration in slices 1, 3 and 7. **(C)** Ten cellular niches identified based on the cellular composition ratio of spatial transcriptomic spots, among which Niche 5 is a unique niche composed of PTPRR−high epithelial cells and iCAFs, localized to PTPRR−high regions. **(D)** Cellular composition and proportion of the niche. **(E)** Spatial distribution of 17 molecular niches identified after batch effect removal across samples. **(F)** Seventeen molecular niches identified after batch effect correction. **(G)** KEGG pathway enrichment analysis of differentially expressed genes in molecular niche Cluster 5, with a total of 8 pathways enriched, including tight junction, focal adhesion and endocytosis, which are related to cell−cell communication. **(H)** Co−localization pattern of iCAFs and PTPRR−high epithelial cells in the GSE235449 spatial transcriptome, showing preferential co−localization of a small number of iCAFs with PTPRR−high epithelial cells.

To explore the association between PTPRR−high epithelial cells and the tumor microenvironment, CAFs were subdivided into iCAF, myCAF, and apCAF subsets via gene set scoring (**Supplementary Figure 1H**). The curated signature gene sets used for CAF subtype scoring are listed in detail in **Supplementary Table 2** (32). Annotated single-cell data were further mapped to spatial transcriptomic slices. The proportion of PTPRR−high epithelial cells was significantly higher in slices 1, 3, and 7 than in other slices, with a maximum proportion below 0.4 (**Figure 5A**), further corroborating the link between PTPRR overexpression and pancreatic cancer metastasis.

Notably, although pancreatic cancer is generally a “cold tumor” with low T−cell infiltration, CD8+ T-cell infiltration was nearly absent in these three PTPRR-high slices (**Figure 5B**), showing significant spatial co-localization of high PTPRR regions with low CD8+ T-cell distribution. Cellular niche analysis identified 10 cellular niches (**Figures 5C, D**), among which Niche 5 was unique to PTPRR−high epithelial cells and mainly composed of iCAFs and PTPRR−high epithelial cells. This niche was localized to PTPRR−high regions in slices 1, 3, and 7 (**Figure 5C**), indicating specific interactions between the two cell types.

Spot−level molecular niche analysis after batch correction identified 17 molecular niches (**Figures 5D, F**), among which Molecular Niche 5 was highly spatially consistent with Cellular Niche 5. Differential gene and KEGG enrichment analyses of this niche revealed eight enriched pathways, including tight junctions, focal adhesions, and endocytosis, all related to cell−cell communication (**Figure 5G**). Further spatial localization showed that a small subset of iCAFs preferentially co−localized with PTPRR−high epithelial cells rather than extensive co−localization (**Figure 5H**), suggesting specific crosstalk prior to metastasis that provides a microenvironmental basis for the metastatic potential of PTPRR−high epithelial cells.

### 3.5 Cell-cell communication characteristics and molecular mechanisms of PTPRR-high epithelial cells

**Figure 6:**
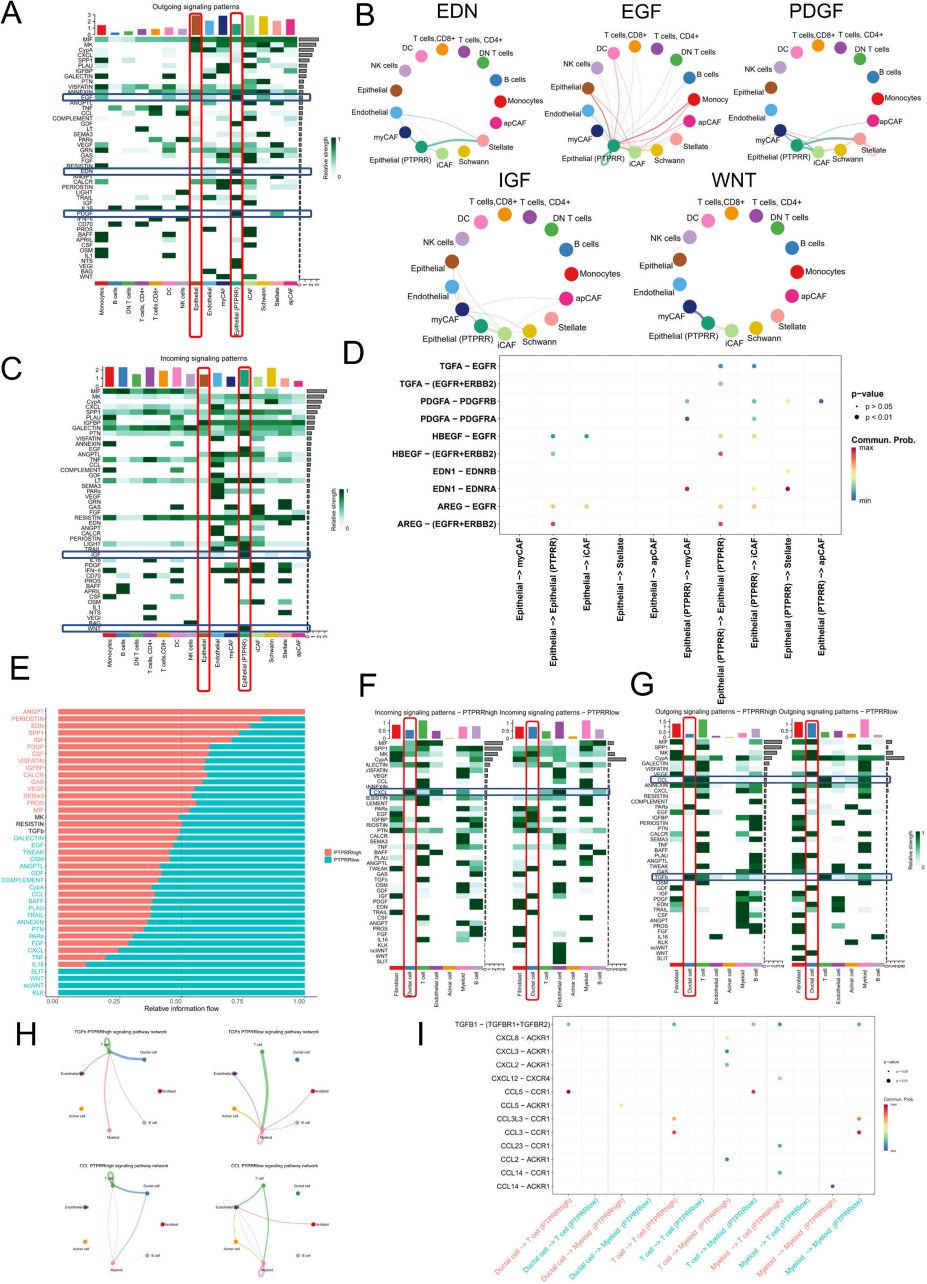
Cell−cell communication characteristics and immunosuppressive pathway analysis of PTPRR−high epithelial cells and samples. **(A, B)** Heatmaps of CellChat analysis in the GSE194247 single−cell dataset, showing the signal output **(A)** and input **(B)** strength of the PTPRR−high epithelial cell subset with other cell clusters. **(C)** Circle plot of signal communication between the PTPRR−high epithelial cell subset and CAFs in the GSE194247 dataset, showing that EDN, EGF and PDGF pathways are transmitted from PTPRR−high epithelial cells to CAFs, while IGF and WNT pathways are regulated by iCAFs and myCAFs. **(D)** Core ligand−receptor pairs mediating communication between PTPRR−high epithelial cells and iCAFs in the GSE194247 dataset (TGFA−EGFR, PDGFA−PDGFRB/PDGFRA, EDN1−EDNRA). **(E)** Comparison of overall EDN, IGF and PDGF pathway activities between PTPRR high− and low−expression samples in the GSE263733 dataset. F−G. Differences in signal input **(F)** and output **(G)** between PTPRR high− and low−expression samples in the GSE263733 dataset, showing significantly enhanced CXCL, TGFβ and CCL signaling in PTPRR−high samples. **(H)** Dissection of target cells for TGFβ and CCL signaling in the GSE263733 dataset, showing enhanced TGFβ signaling reflected by strengthened regulation of T cells by ductal cells and T−cell autocrine signaling, and enhanced CCL signaling reflected by strengthened regulation of T cells and myeloid cells by ductal cells. **(I)** Heatmap of ligand‑receptor interaction pairs among ductal cells, T cells, and myeloid cells in PTPRR high and PTPRR low groups.

Based on the spatial co−localization of PTPRR−high epithelial cells and iCAFs revealed by spatial transcriptomics, we further performed cell−cell communication analysis to clarify the molecular mechanisms of their interaction. CellChat analysis of the GSE194247 single−cell dataset focused on the PTPRR−high epithelial subset, with heatmaps showing signal output and input strength between this subset and other cell clusters (**Figures 6A, B**). In primary pancreatic cancer samples, the PTPRR−high epithelial subset exhibited significantly elevated activity of the EDN, EGF, and PDGF pathways compared with conventional epithelial cells. Circle plot visualization confirmed that these three pathways were mediated by PTPRR−high epithelial cells acting on CAFs (**Figure 6C**), with core ligand−receptor pairs regulating iCAFs including TGFA−EGFR, PDGFA−PDGFRB/PDGFRA, and EDN1−EDNRA (**Figure 6D**). Meanwhile, the PTPRR−high epithelial subset showed enhanced IGF and WNT signal output, mainly regulated by iCAFs and myCAFs (**Figure 6C**), further supporting close crosstalk between PTPRR−high epithelial cells and iCAFs.

To compare the tumor microenvironment between PTPRR high− and low−expression samples, grouped cell−cell communication analysis was performed on the GSE263733 dataset, focusing on global immune microenvironment features. PTPRR−high samples (mostly metastatic) showed slightly increased activity of EDN, IGF, and PDGF pathways compared with PTPRR−low samples (mostly primary) (**Figure 6E**). Further comparison of signal input and output revealed significantly enhanced CXCL signaling and upregulated TGFβ and CCL signal output in PTPRR−high samples (**Figures 6F, G**), all of which are closely related to immunosuppression.

Mechanistic dissection showed that enhanced TGFβ signaling was reflected in strengthened regulation of T cells by ductal (PTPRR−high epithelial) cells and autocrine regulation of T cells, while enhanced CCL signaling was characterized by increased regulation of T cells and myeloid cells by ductal cells, along with elevated T−cell autocrine signaling **Figure 6H**. Ligand−receptor pair analysis identified TGFB1−(TGFBR1+TGFBR2) and CCL5−ACKR1 as key pairs mediating T−cell regulation, both canonical immunosuppressive ligand−receptor pairs. Additionally, CXCL chemokines on T cells can act on ACKR1−expressing immune cells, further reinforcing the immunosuppressive microenvironment **Figure 6I**. These results demonstrate that the core difference in immune microenvironment between PTPRR high− and low−expression samples lies in the regulatory effect of PTPRR−high ductal cells on immune cells, constructing an immunosuppressive microenvironment favorable for pancreatic cancer metastasis—a key molecular mechanism by which PTPRR promotes malignant progression of pancreatic cancer.

## 4 Discussion

Through integrated multi-omics and multi-dataset analysis, this study systematically elucidates the expression pattern and putative oncogenic characteristics of PTPRR in pancreatic cancer at the global, cellular, and spatial levels, filling the gap in PTPRR research in pancreatic cancer. Compared with previous studies using single datasets or single analytical dimensions, this study integrates bulk, single-cell, and spatial transcriptomic data, not only defining the epithelial-specific expression pattern of PTPRR but also revealing its enrichment in metastatic lesions and spatial crosstalk with iCAFs in the tumor microenvironment, greatly enhancing the reliability and comprehensiveness of the conclusions.

Protein tyrosine phosphatase receptor type R (PTPRR), a key member of the receptor−type protein tyrosine phosphatases (RPTPs) family, exerts its core function by regulating downstream signaling pathways through specific dephosphorylation, participating in diverse physiological processes including cell proliferation, differentiation, and migration (6). In recent years, the role of PTPRR in tumorigenesis and progression has attracted increasing attention; however, existing studies indicate significant tumor type−dependent functional heterogeneity. In most malignant tumors, PTPRR acts as a tumor suppressor mainly by inhibiting core oncogenic pathways such as MAPK and PI3K/AKT, and its silenced expression is often mediated by epigenetic modifications including DNA methylation and histone deacetylation (7, 18, 19). In contrast, this multi−dataset and multi−omics integrated study demonstrates that PTPRR exhibits a potential oncogenic phenotype in pancreatic cancer with distinct expression and functional characteristics.

First, single−cell and spatial transcriptomic data confirm that PTPRR is almost exclusively highly expressed in pancreatic ductal epithelial cells (the cell of origin of PDAC) with minimal expression in stromal cells such as immune cells and fibroblasts. This epithelial−specific expression pattern serves as a molecular marker for the ductal epithelial origin of PDAC and reflects the malignant biological features of excessive proliferation and dedifferentiation of tumor cells (20–22). Second, PTPRR expression and the proportion of PTPRR−positive cells are significantly higher in metastatic pancreatic cancer lesions than in primary tumors. TCGA−PAAD analysis identifies PTPRR as a DEG associated with lymph node metastasis and increased tumor volume, and *in vitro* functional assays confirm that PTPRR overexpression significantly promotes pancreatic cancer cell migration and invasion, while its knockdown effectively suppresses malignant phenotypes, indicating that PTPRR is deeply closely associated with pancreatic cancer invasion and metastasis. Importantly, survival analysis reveals that high PTPRR expression is an independent risk factor for poor prognosis in pancreatic cancer patients, with its single−gene expression level effectively predicting overall survival, highlighting its potential as a prognostic biomarker for pancreatic cancer—this is in sharp contrast to the pattern in most cancers where low PTPRR expression predicts poor prognosis (7, 18, 19).

The functional conversion of PTPRR from a tumor suppressor to an oncogene in pancreatic cancer may be primarily driven by the unique genetic background of pancreatic cancer: KRAS mutation (23). Based on the multi-omics correlation analysis in this study, we propose a hypothetical model of KRAS mutation-mediated PTPRR functional conversion; however, due to the lack of direct comparative experiments between KRAS-mutant and wild-type cells, this mechanism remains a scientific hypothesis rather than a conclusive conclusion. KRAS mutation is the most prominent molecular feature of pancreatic cancer, with activating KRAS mutations (mainly G12D and G12V) present in approximately 90% of PDAC patients. Sustained activation of mutant KRAS reshapes the intracellular signaling network and alters the regulatory mode and functional direction of downstream molecules (24–26). Under wild-type KRAS conditions, PTPRR negatively regulates the MAPK pathway by specifically dephosphorylating ERK1/2, inhibiting abnormal cell proliferation and exerting tumor-suppressive effects. In the context of mutant KRAS, constitutively GTP-bound mutant KRAS activates the downstream RAF/MEK/ERK pathway, maintaining ERK1/2 in a hyperphosphorylated state. This aberrantly activated signaling environment induces functional remodeling of PTPRR, converting it from a tumor suppressor to an oncogene and being associated with pancreatic cancer progression (27–30).

Current data only confirm that high PTPRR expression is significantly associated with immunosuppressive microenvironment and pancreatic cancer metastasis, and has not been proven to directly drive immunosuppression through functional experiments. Therefore, PTPRR is temporarily defined as a characteristic biomarker related to pancreatic cancer metastasis, rather than a proven immunosuppressive driver, and whether it is an immunosuppressive driver or merely a passenger marker still needs to be verified by subsequent functional experiments.

We propose the following scientific hypotheses for the upstream regulatory mechanism of specific high expression of PTPRR in metastatic lesions:

1) Copy number variation (CNV): Copy number gain occurs at the PTPRR gene locus in metastatic lesions, leading to up-regulation of transcription level;
2) DNA methylation: Different from the hypermethylation and silencing of PTPRR promoter in other tumors, the PTPRR promoter region in pancreatic cancer metastatic lesions is hypomethylated, thus relieving transcriptional inhibition;
3) Transcription factor regulation: Transcription factors such as AP-1 and ETS families downstream of mutant KRAS can directly bind to the PTPRR promoter region and activate its transcription expression.

These hypotheses provide directions for further in-depth analysis of the transcriptional regulatory mechanism of PTPRR in pancreatic cancer metastasis.

Furthermore, this study finds that high PTPRR expression is more prevalent in metastatic pancreatic cancer patients, whose tumor microenvironment exhibits a distinct immunosuppressive phenotype. Although the direct causal relationship between high PTPRR expression and immunosuppression remains to be determined, PTPRR can be used as a characteristic marker for targeted intervention of related immunosuppressive molecules, providing a novel strategy for pancreatic cancer immunotherapy (30, 31).

The main limitations of this study should be acknowledged: First, most conclusions are based on bioinformatic analysis of public datasets and *in vitro* functional assays, lacking *in vivo* animal model verification to confirm the role of PTPRR in pancreatic cancer metastasis. Second, the hypothetical model of KRAS mutation-mediated PTPRR functional conversion lacks direct experimental evidence, such as comparative experiments between KRAS-mutant and wild-type isogenic cell lines and rescue assays. Third, the upstream regulatory hypotheses of PTPRR high expression (CNV, DNA methylation, transcription factor regulation) have not been experimentally validated. Fourth, the specific molecular mechanism underlying the spatial association between PTPRR and CD8+ T cell infiltration remains unclear.

Correspondingly, future research will focus on the following directions: 1. Construct KRAS wild-type/mutant isogenic cell lines and *in vivo* xenograft models to verify the functional conversion of PTPRR and its role in metastasis; 2. Perform targeted experiments to validate the upstream regulatory mechanism of PTPRR, such as CNV detection, methylation sequencing, and chromatin immunoprecipitation (ChIP) assays for AP-1/ETS transcription factors; 3. Conduct co-culture experiments of tumor cells and immune cells to clarify the potential relationship between PTPRR and immunosuppression; 4. Expand the sample size to further confirm the prognostic value of PTPRR in pancreatic cancer.

## 5 Conclusion

This study is the first to suggest that PTPRR exhibits a potential oncogenic phenotype characterized by epithelial-specific high expression in pancreatic cancer, and its overexpression is closely associated with metastasis and poor prognosis.Signaling network remodeling associated with KRAS mutation represents a hypothetical mechanistic basis for the potential functional switch of PTPRR from a tumor suppressor to an oncogene. This study not only enriches research on the functional heterogeneity of PTPRR across different cancers but also provides novel potential targets and a theoretical foundation for prognostic evaluation and molecular targeted therapy of pancreatic cancer.

## Data Availability

The original contributions presented in the study are included in the article/**Supplementary Material**. Further inquiries can be directed to the corresponding author.

## Supplementary material

Supplementary Figure 1 Survival Analysis Related to Clinical Characteristics of Pancreatic Cancer Patients. **(A)** Kaplan−Meier survival curves for overall survival of pancreatic cancer patients with large vs. small tumor volumes based on the TCGA−PAAD dataset, showing shorter overall survival in patients with larger tumors. **(B)** Supplementary visualization of sample grouping and cell clustering in the GSE263733 single−cell dataset. **(C, D)** Validation of PTPRR expression distribution in the GSE154778 single−cell dataset, showing predominant high expression in pancreatic cancer cells with minimal expression in other cell types. **(E)** Statistics of PTPRR-positive cell proportions in each sample from the GSE263733 dataset. Samples with a positive rate >10% were defined as PTPRR-high samples, which were almost all metastatic samples. Threshold sensitivity analysis with 5%, 10%, 15% and 20% cutoffs was further conducted to validate the robustness of the grouping strategy, and the 10% cutoff was confirmed as the optimal threshold for classification. **(F, G)** Analysis for identifying the PTPRR−high epithelial cell subset by clustering in the GSE194247 single−cell dataset. **(H)** Visualization of CAF subtyping into iCAFs, myCAFs and apCAFs by gene set scoring in the GSE194247 single−cell dataset.

Supplementary Figure 2 Spatial Projection Distribution of apCAF, B cells, DCs, DNT cells, endothelial cells, epithelial cells, iCAF and monocytes in GSE235449 Dataset.

Supplementary Figure 3 Spatial Projection Distribution of myCAF, stellate cells, schwann cells and CD4^+^ T cells in GSE235449 Dataset.

Supplementary Figure 4 Expression profiles and prognostic value of the remaining 14 candidate genes excluding PTPRR.
