# Supplementary figures and images for: Single-cell and spatial transcriptomic profiling reveals the expression characteristics of PTPRR in epithelial cells and its potential implications in pancreatic cancer metastasis
Source: Front Immunol. 2026 May 13;17:1843505. doi: 10.3389/fimmu.2026.1843505 (PMC13212452; doi:10.3389/fimmu.2026.1843505)

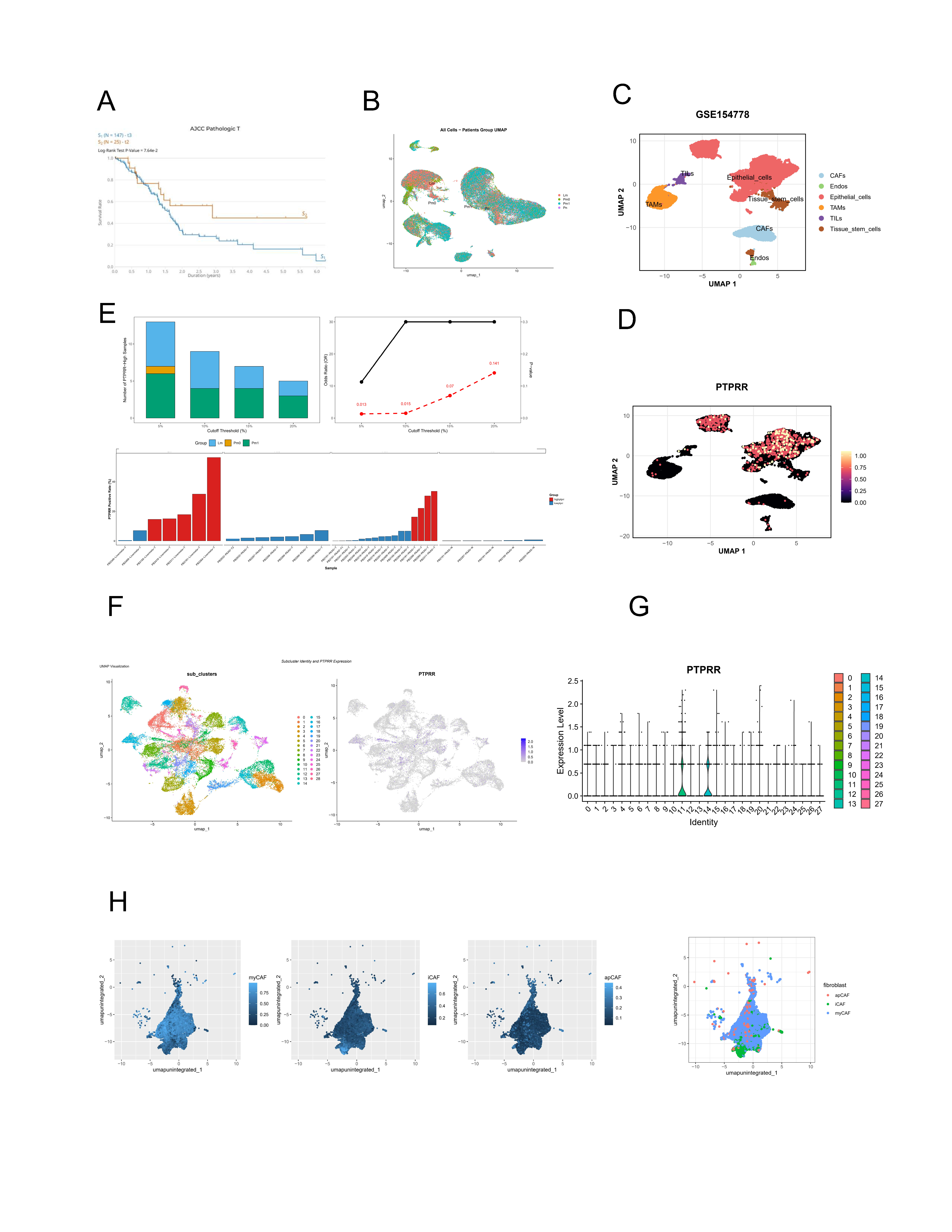

Supplement: Supplementary Figure 1 — Survival Analysis Related to Clinical Characteristics of Pancreatic Cancer Patients. (A) Kaplan−Meier survival curves for overall survival of pancreatic cancer patients with large vs. small tumor volumes based on the TCGA−PAAD dataset, showing shorter overall survival in patients with larger tumors. (B) Supplementary visualization of sample grouping and cell clustering in the GSE263733 single−cell dataset. (C, D) Validation of PTPRR expression distribution in the GSE154778 single−cell dataset, showing predominant high expression in pancreatic cancer cells with minimal expression in other cell types. (E) Statistics of PTPRR-positive cell proportions in each sample from the GSE263733 dataset. Samples with a positive rate >10% were defined as PTPRR-high samples, which were almost all metastatic samples. Threshold sensitivity analysis with 5%, 10%, 15% and 20% cutoffs was further conducted to validate the robustness of the grouping strategy, and the 10% cutoff was confirmed as the optimal threshold for classification. (F, G) Analysis for identifying the PTPRR−high epithelial cell subset by clustering in the GSE194247 single−cell dataset. (H) Visualization of CAF subtyping into iCAFs, myCAFs and apCAFs by gene set scoring in the GSE194247 single−cell dataset. [file Image1.tif]

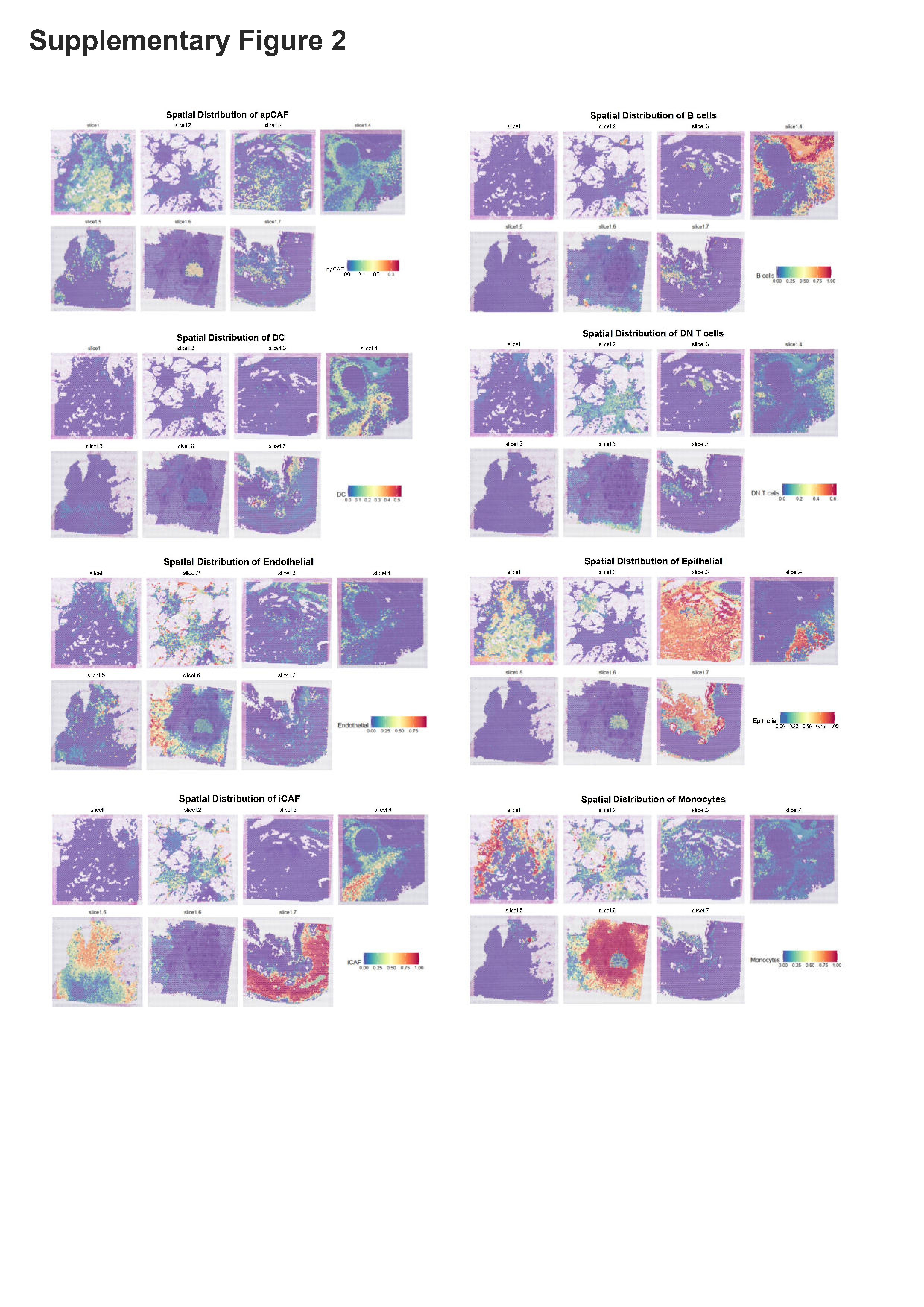

Supplement: Supplementary Figure 2 — Spatial Projection Distribution of apCAF, B cells, DCs, DNT cells, endothelial cells, epithelial cells, iCAF and monocytes in GSE235449 Dataset. [file Image2.tif]

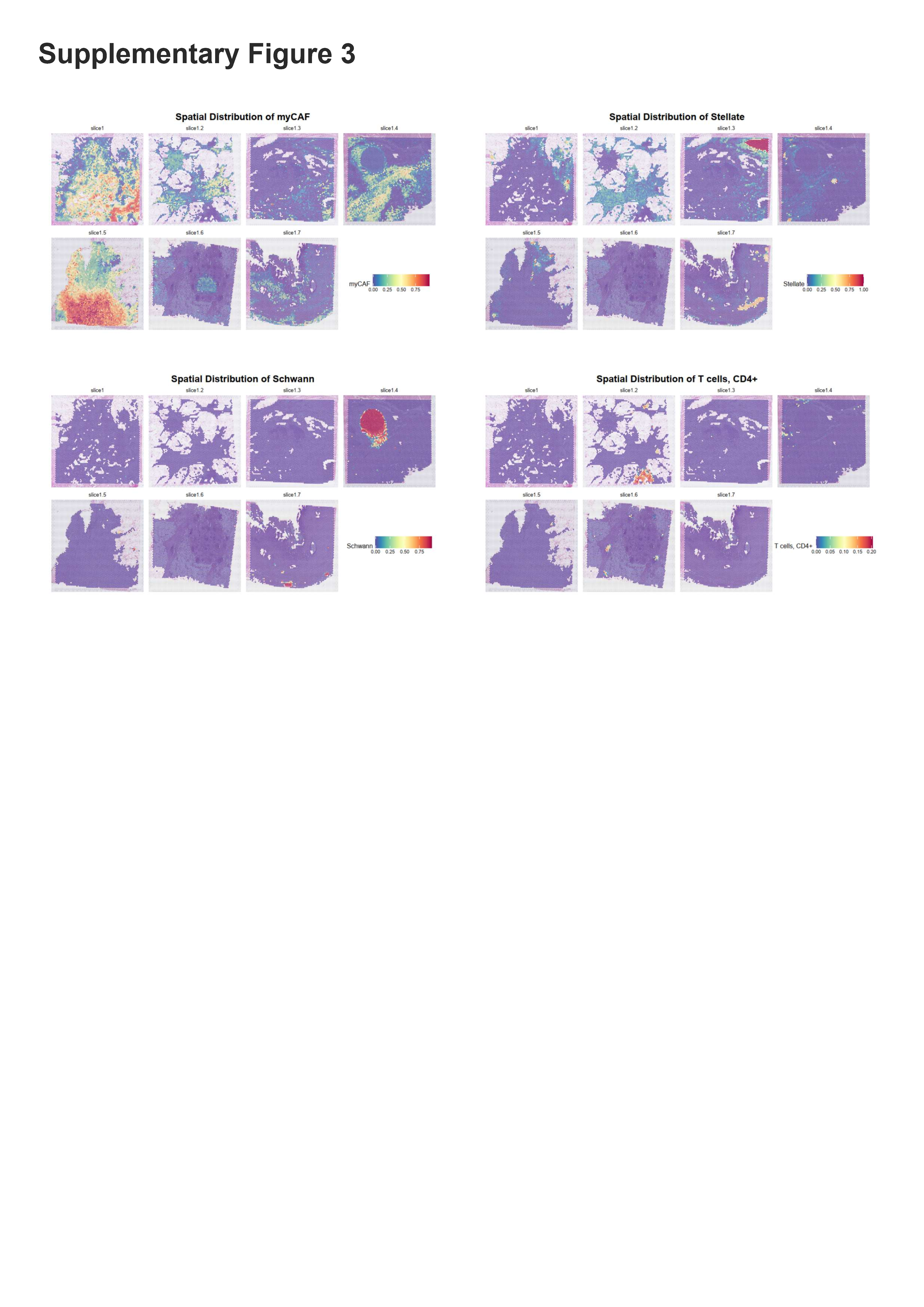

Supplement: Supplementary Figure 3 — Spatial Projection Distribution of myCAF, stellate cells, schwann cells and CD4+ T cells in GSE235449 Dataset. [file Image3.tif]
